# Editorial: Spelling Across Orthographies

**DOI:** 10.3389/fpsyg.2021.700604

**Published:** 2021-06-10

**Authors:** Teresa Limpo, Naymé Salas, Marie Van Reybroeck, São Luís Castro

**Affiliations:** ^1^Faculty of Psychology and Education Sciences, University of Porto, Porto, Portugal; ^2^Departament de Didàctica de la Llengua i la Literatura, i de les Ciències Socials, Facultat de Ciències de l'Educació, Universitat Autònoma de Barcelona, Barcelona, Spain; ^3^Delilab, Psychological Sciences Research Institute (IPSY), UCLouvain, Louvain-la-Neuve, Belgium

**Keywords:** spelling, misspelling analysis, morphology, cross-language comparisons, handwriting

The ability to spell words correctly is a cornerstone of literacy. Despite the substantial amount of research into this process, a large part of the empirical findings come from English-speaking populations. Given the distinctive features of the varying orthographic systems worldwide, more research into spelling across orthographies seems warranted. To stimulate this was the main goal of this Research Topic, which contains two review plus 14 research articles tapping 11 orthographies (viz., Arabic, Catalan, Chinese, English, French, Hebrew, Malay, Portuguese, Spanish, Tamil, and Welsh), from first graders to undergraduates. These articles were organized into three sections focusing on (1) the type of misspellings produced, (2) the role of non-phonological knowledge in spelling, and (3) the view of spelling as a basic writing process. Together, the findings from the studies included in the Research Topic showed that the type of misspellings produced is influenced by writing systems, writers' characteristics, and spelling tasks; that morphological, orthographic, morpho-orthographic, and syntactical knowledge are important sources of information to produce accurate spellings in varying orthographic systems; and that spelling is a fundamental writing process intertwined with handwriting. In sum, this Research Topic provides an up-to-date view on spelling across orthographies, which will contribute to increase our understanding of this process and instigate further research into it.

## Editorial on the Research Topic: Spelling Across Orthographies

Spelling—or the retrieval, assembling, and selection of orthographic symbols—is a fundamental process underlying reading and writing (Graham and Santangelo, 2014). Reflecting the central role of spelling in literacy, a large body of works having spelling as its central object of study has been built (Treiman, 2017). Still, the majority of conclusions emerging from this research is grounded on findings from English-speaking populations. Notwithstanding the importance of those results, a deeper understanding of spelling acquisition and development calls for a broader approach, capable of supporting inferences on the similarities and differences across orthographies. This acknowledgment was the motivating force to set up this collection of articles.

This Research Topic contains two review articles plus 14 articles reporting empirical studies, which targeted spelling across 11 orthographies (viz., Arabic, Catalan, Chinese, English, French, Hebrew, Malay, Portuguese, Spanish, Tamil, and Welsh). Together, these studies covered a large age span, from first-grade school children to undergraduate students. Articles are organized into three sections: section The Informative Nature of Misspellings looks into the type of misspellings produced by children and adolescents across orthographies and tasks; section Non-phonological Sources of Knowledge explores the non-phonological knowledge sources involved in spelling across varying writing systems; and section Spelling as a Basic Writing Process approaches spelling as a basic writing process, related to handwriting and important to produce good texts.

### The Informative Nature of Misspellings

In the last decades, researchers have developed several spelling scoring methods, which are fine-grained alternatives to the traditional correct/incorrect scoring (Treiman et al., 2019). These methods provide detailed information about the challenges imposed by spelling in varying writing systems, different developmental points, or writing tasks with variable demands.

Five articles included in this section of the Research Topic focused on the types of misspellings produced by writers, among which three compared different orthographies. Joye et al. examined error types produced in dictation and composition by monolingual 9–10-year-olds with Developmental Language Disorder, speakers of French and English. Findings revealed more morphological errors in French than English in both tasks and more orthographic errors in English than French in the dictation task. Additionally, segmentation and contraction errors were more frequent in French, whereas morphological ending errors appeared more often in English. O'Brien et al. studied phonological, orthographic, and morphological errors across three language groups composed of bilingual children learning English plus an Asian alphabetic script (Malay), akshara script (Tamil), or hanzi script (Mandarin Chinese). Results showed that the Tamil group produced more overall errors and that the three groups differed in the proportions of phonological errors (more prevalent in Malay) and morphological errors (more prevalent in Malay and Chinese, albeit rare). In older, adult participants Martin et al. explored differences in spelling and phonological awareness between distinctive writing systems. Authors compared L1 English speakers with speakers of English as a second language (ESL) using different L1 writing systems: alphabet, abjad, and morphosyllabary. L1 English speakers performed better than all ESL groups, which differed in terms of their performance: the morphosyllabic L1 group showed the highest word spelling accuracy and very low pseudoword spelling accuracy; the alphabetic L1 group showed the lowest spelling and phonological awareness accuracy. The misspellings analysis revealed vowels to be more problematic than consonants, particularly in abjad L1 speakers.

Two other articles provided a comparison of error types between grades within the same orthographic system. Magalhães et al. examined Portuguese children's misspellings across grade (2, 4, and 6), type (phonetically inaccurate, phonetically accurate, and stress mark errors), and task (dictation and composition). Results showed a progressive decrease in all error types except stress mark errors, which were more frequent in Grade 4; and more misspellings in dictation than composing tasks. Spelling errors were found to be associated with texts of worse quality. Yassin et al. tested the impact of visual-orthographic features of the Arabic abjad on spelling errors produced by first, second, and fourth graders. Results showed a high rate of errors across all grades, with visual-orthographic spelling errors accounting for over one quarter of these. This category of errors was ranked the second most frequent one, below violations of spelling conventions and above phonological errors.

### Non-phonological Sources of Knowledge

Despite the undeniable role of phonology in spelling, there is now a substantial amount of evidence showing that non-phonological sources of knowledge are used to spell words correctly from very early on (Treiman, 2017). Some of these sources are explored in this section, across six articles.

Salas looked into the non-phonological spelling strategies used by Catalan-speaking children in Grade 2 and 4, with exposure to Catalan outside school or not. Results were similar regardless of Catalan exposure and showed that strategies requiring morphophonological or orthographic knowledge were mastered before those requiring morphological or lexical knowledge. Moreover, all non-phonological strategies had a significant and unique contribution to conventional spelling. In a sample of Portuguese children, Vale and Perpétua also showed the reliance on non-phonological information from very early on, by examining the spelling of the schwa (/ə/)—a phonologically (or minimal) segment—absent in Portuguese first graders at two time points, with a 3-month gap. Despite the weak alphabet knowledge at the first assessment, children tended to represent schwa vowels mostly with the appropriate letter <e>. This representation increased over 3 months and, at both time points, was used more often in potentially orthographic illegal than legal phonological consonantal clusters.

Two additional articles present cross-sectional studies investigating morphological-related knowledge. Schiff et al. focused on the role of morpho-orthographic principles in homophonous affix letter spelling among Hebrew speaking students in Grades 2, 4, 7, and 10. Despite the increased accuracy across all affix letters, findings showed a differential application of morpho-orthographic principles throughout schooling. Younger spellers were mostly assisted by morpho-orthographic sites, morphological category frequency, and phonological transparency, whereas the spelling of older ones was more affected by morpho-orthographic prevalence. Mussar et al. explored morphological knowledge in a cross-sectional study with French-speaking children in Grades 1, 2, and 3. Results showed that children's performance on four morphological knowledge tasks improved across grades, even though they struggled more with explicit than implicit tasks. Moreover, those tasks converged into a single morphological knowledge factor that predicted children's ability to represent words with silent-letter endings, after controlling for grade, reading for pleasure, and general orthographic word recognition.

The definition of morphological spelling is however not consensual, as discussed in the review article of Weth, who proposes that syntactic markers (e.g., inflectional suffixes) should be distinguished from morphological spelling, which considers inflection only in relation to the orthographic word. On the contrary, syntactic markers seem a specific category that is part of the orthographic word but also indicate relational information on phrase and clause level. Highlighting the need to examining spelling by questioning the knowledge of grammatical categories required to choose the correct spelling, Van Reybroeck conducted a study in 9–12-year-old French-speaking children with dyslexia that aimed to understand their grammatical spelling difficulties. Compared to grammatical spelling and age-matched peers, children with dyslexia identified fewer subjects of different complex-structure sentences, suggesting a specific deficit in syntactic awareness.

### Spelling as a Basic Writing Process

As proposed in many cognitive models of writing (e.g., Graham, 2018), the activity of producing text relies on the enactment of several processes. Together with handwriting (or typing), spelling constitutes a very basic writing process (i.e., transcription). The link between spelling and writing processes is addressed in this section.

Caravolas et al. compared the spelling and handwriting legibility of Welsh-English bilingual children in Grades 3–5 with same age and same spelling-ability English-monolingual peers. As expected, bilingual children displayed weaker spelling and handwriting skills than age-matched peers. A major finding was that handwriting legibility improved more with spelling ability than with handwriting practice emerging from years of schooling and maturation. Ding et al. investigated the links between handwriting fluency and spelling accuracy in a 2-year longitudinal study that followed children living in mainland China from the third to the fifth grade. Cross-lagged analysis showed a bidirectional predictive association between handwriting and spelling, after accounting for the well-established cognitive measures. Suárez-Coalla et al. investigated how the spelling deficits associated with dyslexia affect the dynamics of handwriting in 9–12-year-old native Spanish speakers. Compared to their chronological age-matched peers, children with dyslexia showed longer writing durations, a larger effect of word frequency in within-word pauses in articles and nouns, and a more prolonged phonology-to-orthography consistency effect in the pauses before the target word.

Providing a broader perspective on the role of spelling in literacy, Llauradó and Dockrell explored the relationships between handwriting, spelling, reading, and text production among second, fourth, and sixth graders speaking three different languages: Catalan, English, and Spanish. Spanish children produced fewer misspellings and spelling ability did not predict text quality. Though both English and Catalan children were challenged by spelling, their ability to spell correctly only influenced text quality in English. Evidence on the central role of spelling in the development of solid literacy skills has motivated the development of several instructional programs. Among these, technology-mediated ones have been gaining prominence, such as the GraphoLearn technology. This is reviewed by Lyytinen et al. with a focus on its effectiveness to the acquisition of basic spelling skills in different alphabetic writing systems, mainly in Occidental countries. The use of a game-based technology to support the teaching of reading and writing in Asia and Africa is also discussed.

## Conclusion

This Research Topic gathered a collection of articles dealing with issues related to spelling in several orthographic systems. Our ultimate goal was to intensify discussions about the specific and universal underpinnings of spelling acquisition and development. Several insightful discussions had already taken place during the elaboration of this work. We do hope those reflections will continue and stimulate new research into spelling. This will deepen our knowledge about spelling and, ultimately, promote its acquisition, and development around the globe.

## Author Contributions

TL wrote a first draft of the manuscript. All authors reviewed and approved the manuscript.

## Conflict of Interest

The authors declare that the research was conducted in the absence of any commercial or financial relationships that could be construed as a potential conflict of interest.
